# F-box protein FBXO22 mediates polyubiquitination and degradation of KLF4 to promote hepatocellular carcinoma progression

**DOI:** 10.18632/oncotarget.4082

**Published:** 2015-05-28

**Authors:** Xin Tian, Shundong Dai, Jing Sun, Guojiang Jin, Shenyi Jiang, Fandong Meng, Yan Li, Di Wu, Youhong Jiang

**Affiliations:** ^1^ Molecular Oncology Laboratory of Cancer Research Institute, The First Affiliated Hospital of China Medical University, Shenyang, 110001, China; ^2^ Department of Pathology, The First Affiliated Hospital and College of Basic Medical Sciences of China Medical University, Shenyang, 110001, China; ^3^ Institute of Pathology and Pathophysiology, Shenyang, 110001, China; ^4^ Department of Immunology and Biotherapy, Liaoning Cancer Hospital and Institute, Shenyang, 110042, China; ^5^ Department of Laboratory Medicine, The First Affiliated Hospital of China Medical University, Shenyang, 110001, China; ^6^ Department of Rheumatology, The First Affiliated Hospital of China Medical University, Shenyang, 110001, China

**Keywords:** Kruppel-like factor 4, FBXO22, hepatocellular carcinoma, ubiquitination

## Abstract

Kruppel-like factor 4 (KLF4), a member of the KLF family of transcription factors, has been considered as a crucial tumor suppressor in hepatocellular carcinoma (HCC). Using affinity purifications and mass spectrometry, we identified FBXO22, Cullin1 and SKP1 as interacting proteins of KLF4. We demonstrate that F-box only protein 22 (FBXO22) interacts with and thereby destabilizes KLF4 via polyubiquitination. As a result, FBXO22 could promote HCC cells proliferation both *in vitro and in vivo*. However, KLF4 deficiency largely blocked the proliferative roles of FBXO22. Importantly, FBXO22 expression was markedly increased in human HCC tissues, which was correlated with down-regulation of KLF4. Therefore, our results suggest that FBXO22 might be a major regulator of HCC development through direct degradation of KLF4.

## INTRODUCTION

KLF4, a member of the krüppel-like factor (KLF) transcription factor family, is a potential tumor suppressor in several types of human malignancies [1–4]. For instance, KLF4 was significantly down-regulated in prostate cancer cell lines compared with nontumorigenic prostate cells [5]. RNA activation-mediated overexpression of KLF4 inhibited prostate cancer cell proliferation and altered the expression of several downstream cell-cycle-related genes [5]. Besides, ablation of KLF4 in gastric progenitor cells promoted transformation of the gastric mucosa and tumorigenesis in the antrum in mice [6]. Moreover, KLF4 protein expression was decreased or lost in hepatocellular carcinoma (HCC) tissues and, in particular, lymph node metastases when compared with that in normal liver [7]. Deficiency of KLF4 expression was significantly associated with poor survival, and also a prognostic marker in HCC patients [7]. Indeed, KLF4 could inhibit cell proliferation, invasion and epithelial to mesenchymal transition (EMT) through up-regulation of vitamin D receptor (VDR) and repression of β-catenin and SLUG, respectively [7–10]. However, the molecular mechanisms for the down-regulation of KLF4 in HCC tissues remain poorly understood.

It has been shown that KLF4 protein could be regulated by acetylation, phosphorylation and sumoylation [11–14]. More importantly, Lim KH et al. reports that KLF4 undergoes proteasomal degradation and some lysine residues are critical for its ubiquitination [15]. However, the factors regulating the ubiquitination and degradation of KLF4 have not been fully understood.

The Skp1-Cul1-F box protein ubiquitin ligases (SCFs), consisting of Skp1, Cul1, and one of a member of F box proteins, play important roles in various biological events by their ability to bind and destabilize substrates [16, 17]. For instance, FBXW7 could inhibit tumor growth by targeting Cyclin E, c-myc and SRC-3, all of which are thought to be oncoproteins [18–21]. Indeed, FBXW7 mutations have been found in a variety of primary human tumor types, providing novel insights into cancer progression and development of therapeutic drugs [22, 23]. In the current study, we used an immunoprecipitation and tandem mass spectrometry (IP-MS) analyses to search for KLF4-interacting protein with potential ubiquitin ligase activity and identified FBXO22 that binds with KLF4.

## RESULTS

### Identifying FBXO22 as a KLF4-interacting protein

**Figure 1:**
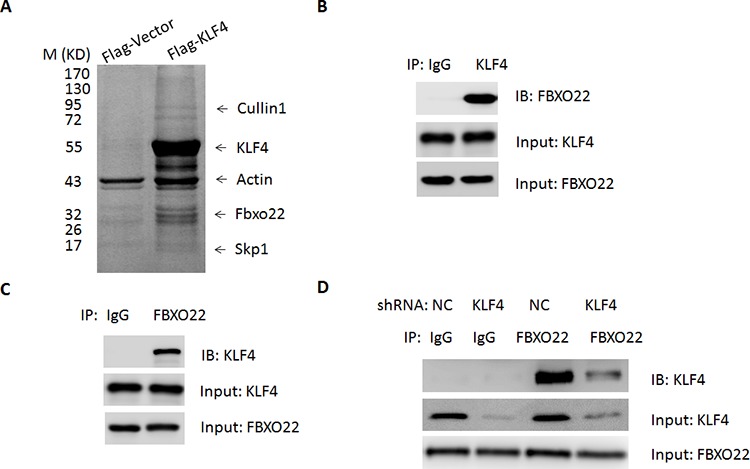
FBXO22 interacts with KLF4 *in vivo* **A.** HepG2Cells were transfected with adenovirus expressing Flag-KLF4 or empty vector for 36 hr. The pull-down products from Flag M2 beads affinity purification were separated by SDS-polyacrylamide gel electrophoresis (SDS-PAGE), visualized by Colloidal blue staining, and analyzed by mass spectrometry. The identified SCF^FBXO22^ components are listed. **B–C.** Endogenous association between FBXO22 and KLF4 in HepG2 cells was performed by co-immunoprecipitation (CO-IP) experiments. **D.** The interaction of FBXO22 and KLF4 in cells depleted with KLF4. HepG2 cells were administered with adenoviral shRNA targeting KLF4 or a negative control (NC)

To identify novel KLF4-interacting proteins, HepG2 cells were transfected with adenovirus expressing Flag-KLF4 or empty vector for 36 hr. Then proteins were isolated by Flag M2 beads, separated by SDS-PAGE, and identified by tandem mass spectrometry (MS/MS). As shown in the Figure 1A, the examination of copurified endogenous proteins revealed the presence of unique peptides derived from SCF subunits, including Cullin1, SKP1 and F-box protein FBXO22 as major KLF4-associated proteins. FBXO22 is of interest since the F-Box protein family has been reported as the largest and most versatile class of E3 ubiquitin ligase. A FBXO22-KLF4 interaction was further confirmed by substantial portion of endogenous FBXO22 and KLF4 co-immunoprecipitated together (Figure 1B and 1C). This interaction was largely attenuated in KLF4-knockdown cells, suggesting the specificity of this interaction (Figure 1D). To confirm the specific binding between KLF4 and FBXO22, we screened 11 human F box proteins. These FLAG tagged F-box proteins were transfected into 293T cells and then immunoprecipitated to evaluate their interaction with endogenous KLF4. We found that, although all F-box proteins could interact with SKP1, only FBXO22 interacts with endogenous KLF4 (Supplementary Figure 1). In contrast, related F box proteins such as FBXO4, FBXO6, FBXO30, FBXO34, or FBXO36, which all belong to F-box only (FBXO) proteins, did not bind endogenous KLF4.

### FBXO22 promotes KLF4 ubiquitination and degradation

**Figure 2:**
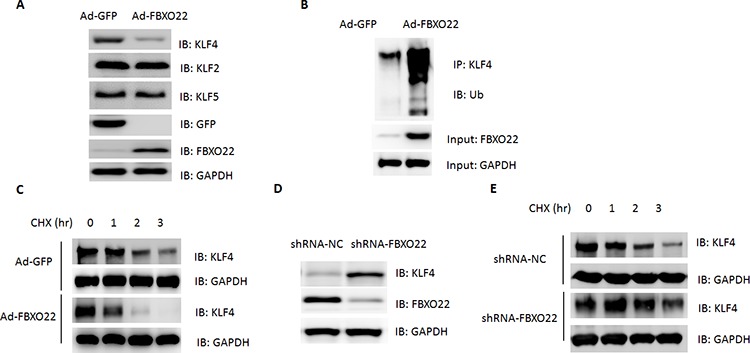
FBXO2 promotes KLF4 turnover in HCC cells **A.** The levels of KLF4, KLF2 and KLF5 in cell lysates were determined by Western blot. HepG2 cells were transfected with adenovirus expressing GFP or FBXO22 for 36 hr. **B.** Ubiquitination of KLF4 in whole-cell lysates was determined by Western blot. **C.** HepG2 cells were transfected with adenovirus expressing GFP or FBXO22. 36 hr after transfection, the cells were either lysed directly or incubated in the presence of cyclohexamide (50 μg/ml) for the indicated time period in order to determine KLF4 turnover. **D.** The levels of KLF4 in cell lysates were determined by Western blot. HepG2 cells were transfected with adenoviral shRNA targeting FBXO22 or a negative control (NC) for 36 hr. **E.** KLF4 turnover was determined by Western blot in HepG2 cells transfected with adenoviral shRNA targeting FBXO22 or a negative control (NC) for 36 hr.

Members of F-Box proteins could recognize and mediate the degradation of target proteins. Thus, the fact that FBXO22 interacts with KLF4 prompted us to ask whether FBXO22 can promote the degradation of KLF4 as well. To test this hypothesis, adenovirus containing FBXO22 or GFP was introduced into HepG2 cells. Forced expression of FBXO22 led to a decreased protein levels of KLF4 (Figure 2A). The effect on KLF4 stability is selective since other members of KLF family (KLF2 and KLF5) were unaffected by FBOXO22 overexpression (Figure 2A). Besides, the reduction in KLF4 protein is not due to changes at the KLF4 mRNA levels, as shown by quantitative real-time PCR (Supplementary Figure 2). The decrease of KLF4 was also observed when FBXO22 was overexpressed in other HCC cells (HuH7 and Hep3B) and normal hepatocytes (L02) (Supplementary Figure 3A–3C). Ubiquitin-conjugation to KLF4 was enhanced by FBXO22 overexpression (Figure 2B). Besides, p21^Cip1/WAF1^ and Cyclin B1, two cell-cycle regulators, were activated and repressed by KLF4 respectively [24, 25] and modulated by FBXO22 (Supplementary Figure 3D–3E).

**Figure 3:**
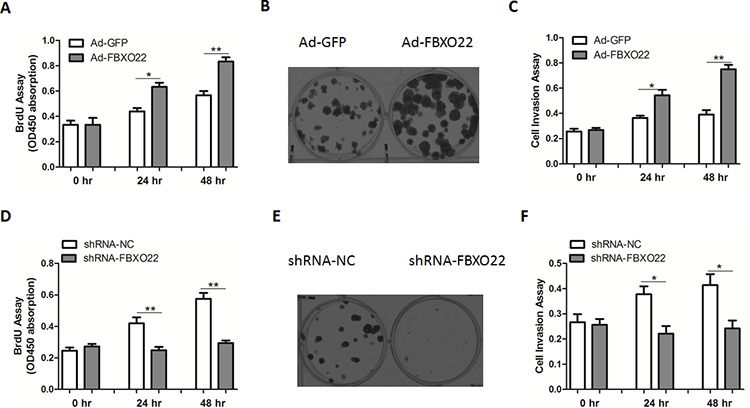
FBXO22 promotes HCC cell proliferation and invasion Cell proliferation **A, D.**, colony formation **B, E.** and invasion **C, F.** assays were determined in HepG2 cells with FBXO22 overexpression or knockdown.

To further analyze the FBXO22-mediated down-regulation of KLF4 in more detail, degradation kinetics of KLF4 was determined in HepG2 cells. As expected, expression of FBXO22 significantly reduced the half-life of KLF4 (Figure 2C). On the other hand, we examined whether knockdown of FBXO22 could inhibit KLF4 turnover. FBXO22 deficiency resulted in an increased expression and half-life of KLF4 (Figure 2D–2E, Supplementary Figure 4A–4C), indicating that FBXO22 contributes to the turnover of KLF4.

### FBXO22 promotes HCC cell proliferation and invasion *in vitro*

**Figure 4:**
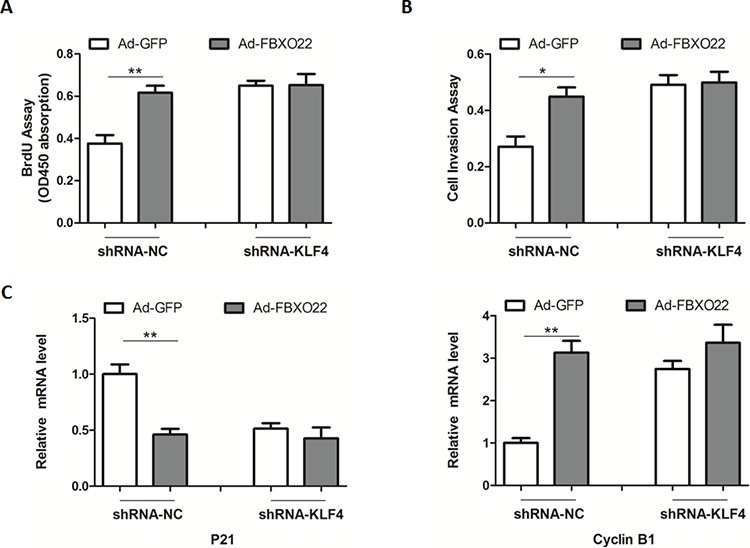
The oncogenic roles of FBXO22 rely on its regulation of KLF4 **A–B.** Cell proliferation (A) and invasion (B) assays in cells with FBXO22 overexpression and (or) KLF4 knockdown as indicated. HepG2 cells were transfected with adenovirus expressing GFP or FBXO22 for 24 hr, and then transfected with adenoviral shRNA targeting KLF4 or a negative control (NC) for another 24 hr. **C.** Relative mRNA levels of p21 and Cyclin B1 in HepG2 cells as in (A-B)

Given that KLF4 appears to function as a tumor suppressor in HCC, we next determined what physiologically stimulates FBXO22 to interact with and stabilize KLF4. As a result, the interaction of KLF4 and FBXO22 was enhanced during the cell-cycle progression, leading to a reduced protein levels of KLF4 (Supplementary Figure 5). Besides, abilities of cell proliferation, colony formation and invasion were significantly enhanced by FBXO22 overexpression (Figure 3A–3C). In contrast, disruption of FBXO22 in HepG2 cells inhibited cell growth (Figure 3D–3F). Additionally, KLF4 deficiency largely blocked the proliferative roles of FBXO22 in HepG2 cells (Figure 4A–4B). In agreement, expression levels of p21^Cip1/WAF1^ and Cyclin B1 inhibited by FBXO22 were also attenuated by KLF4 silencing (Figure 4C), suggesting the oncogenic effects of FBXO22, at least in part, depend on its regulation of KLF4.

### FBXO22 promotes HCC growth *in vivo*

**Figure 5:**
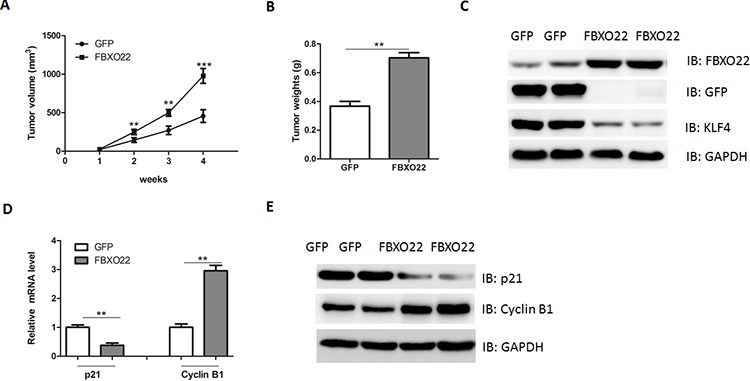
FBXO22 promote HCC growth *in vivo* **A–B.** HepG2 cells stably transfected with FBXO22 or GFP were injected into nude mice and followed up for tumorigenesis. Growth curve of tumor volumes (A) and tumor weights (B) were taken 4 weeks after injection. (*n* = 6–8 for each group) **C.** Representative protein levels of FBXO22, GFP and KLF4 were determined in the two groups of tumors. **D–E.** mRNA and protein levels of p21 and Cyclin B1 in two groups of tumors were determined by real-time PCR and Western blot.

To demonstrate the function of FBXO22 *in vivo*, HepG2 cells stably overexpressing FBXO22 or GFP protein were injected subcutaneously into two bilateral sites of BALB/c nude mice. In agreement, the tumor volume and weight were markedly increased in FBXO22-overexpressed tumors compared to control tumors (Figure 5A–5B). KLF4 protein contents were reduced in FBXO22 overexpressed tumor tissues (Figure 5C), suggesting an oncogenic role of FBXO22 *in vivo*. Consistently, in FBXO22 overexpressed tumor tissues, the expression levels of p21^Cip1/WAF1^, and Cyclin B1 were also affected (Figure 5D–5E).

### Up-regulation of FBXO22 in human hepatocarcinoma

**Figure 6:**
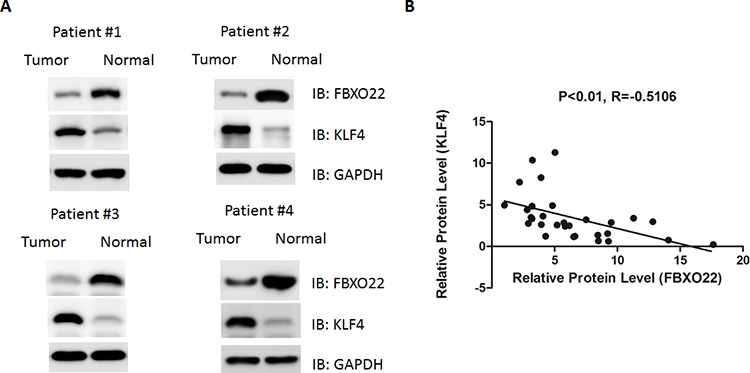
FBXO22 expression was increased in human hepatocarcinoma, correlating with reduced KLF4 expression **A.** Protein levels of FBXO22 and KLF4 were determined by Western blot in human HCC tissues and adjacent normal tissues. **B.** The correlation between FBXO22 and KLF4 protein levels in paired HCC and non-tumor tissues.

Finally, we asked whether FBXO22-mediated KLF4 degradation is involved in human HCC development. As shown in the Figure 6A, the expression of FBXO22 was significantly increased in tumor samples compared with adjacent normal tissue. In contrast, down-regulation of KLF4 was detected in tumor tissues (Figure 6A). Association analysis of 30 HCC tissues indicated that the protein levels of FBXO22 and KLF4 was negatively correlated (Figure 6B), supporting the connection between FBXO22 and KLF4.

It has been well-established that inflammatory process is tightly associated with many different cancer types, including HCC. We speculate that the up-regulation of FBXO22 in HCC tissues might be attributed to aberrant activation of inflammatory signaling. Therefore, HepG2 and HuH7 cells were treated with TNFα and IL-1β. As expected, treatment of these pro-inflammatory cytokines significantly increased the mRNA and protein levels of FBXO22 (Supplementary Figure 6A–6D). These results suggest that the up-regulation of FBXO22 in HCC tissues, at least in part, due to persistent activation of inflammatory signaling.

## DISCUSSION

In the present study, we have demonstrated that KLF4 is a biological target of FBXO22, which promotes the turnover of KLF4 and thereby promotes HCC cell proliferation and invasion. It has been shown that promoter methylation of KLF4 locus could contribute to its down-regulation *in vitro* and *in vivo* [26, 27]. KLF4 was also identified as a direct target of several microRNAs, a class of small and non-coding RNA molecules [28, 29]. Besides, TGF-β signaling could promote KLF4 degradation through Cdh1-anaphase promoting complex [30]. In addition, Phosphorylation of KLF4 by ERK1 or ERK2 enhanced its interaction with the F-box proteins βTrCP1 or βTrCP2, which led to KLF4 ubiquitination and degradation [13]. Because KLF4 is a tumor suppressor clearly involved in the initiation and (or) progression of HCC, it would be reasonable that important regulatory checks on its expression may exist at multiple levels. Therefore, our data present a novel mechanism for the dysregulation of KLF4 in HCC. However, further studies are still needed to establish the connection between FBXO22 and KLF4 in other gastrointestinal cancer.

Tan MK et al. reports that FBXO22 could control the activity of KDM4A through targeting it for proteasomal turnover [31], by which FBXO22 regulates histone H3 lysine 9 and 36 methylation levels in cell cycle. Besides, a recent study indicates that FBXO22 is essential for optimal synthesis of the N-methyl-D-aspartate (NMDA) receptor coagonist D-serine [32]. Here, we provide evidence that FBXO22 is up-regulated and might be an oncogene in HCC. However, the expression and roles of FBXO22 in other types of human cancers remain to be determined.

In conclusion, our study now suggests that FBXO22 controls the turnover of KLF4 protein *in vivo* and *in vitro*. Inhibition or down-regulation of FBXO22 by gene therapy might be beneficial in patients with malignant tumors.

## MATERIALS AND METHODS

### Human tissue samples

30 paired of HCC tissues and adjacent non-tumor normal tissues were collected from routine therapeutic surgery at our department. All samples were obtained with informed consent and approved by the hospital institutional review board.

### Cell culture

HCC cell lines (HepG2, HuH7 and Hep3B cells) were obtained from The Cell Bank of Type Culture Collection of Chinese Academy of Sciences (CAS, Shanghai). Cells were grown in Dulbecco's modified Eagle's medium (DMEM, Gibco, Shanghai) supplemented with 10% fetal bovine serum (Gibco) and maintained at 37°C in a humidified atmosphere with 5% CO_2_.

### Mouse experiments

Male BALB/c nude mice aged 4 weeks were purchased from Shanghai Laboratory Animal Company (SLAC, Shanghai). 4.5 × 10^6^ HepG2 cells stably expressing GFP or FBXO22 were injected subcutaneously to the skin under the front legs of the mouse. The mice were observed over 4 weeks for tumor formation. After the mice were sacrificed, the tumors were recovered and the wet weights of each tumor were determined.

### Immunoprecipitation

The purification process has been reported previously. Briefly, 5 × 10^7^ cells infected with adenovirus expressing Flag-KLF4 or empty vector were lysed in 5 mL of NETN lysis buffer for 20 min on ice. Lysates were cleared using centrifugation at 13000 rpm for 20 min; the supernatant was then subjected to immunoprecipitation with 50 μL of anti-FLAG M2 affinity resin overnight at 4°C with gentle inversion. Resin containing immune complexes was washed with 1 mL ice cold lysis buffer 6 times and proteins were eluted with 150 μL 150 μg/mL 3 × Flag-peptide (Sigma) in TBS for 10 min. Proteins were precipitated with cold acetone.

### In gel tryptic digestion

Immunoprecipitation samples were separated by SDS-PAGE, and visualized with colloidal Coomassie blue. The interest bands lane were cut into 1 mm slices, and each slice was washed twice with 50 mM NH4HCO3, 50% ACN and dehydrated with ACN. Proteins were reduced and alkylated by treating them with 10 mM DTT and 55 mM iodoacetamide, respectively. After washing with 50 mM NH4HCO3 and ACN, proteins were digested in gel with trypsin and incubated overnight at 37°C. Tryptic peptides were extracted from the gel pieces with 60% ACN, 0.1% trifluoroacetic acid. The peptide extracts were vacuum centrifuged to dryness.

Samples were desalted with 10ul U-C18 pipette tips.

### Nano-HPLC-MS/MS Analysis

The dried samples were dissolved in solvent A (0.1% formic acid, 2% acetonitrile, 98% H2O). Samples were then injected onto a manually packed reversed phase C18 column (150 mm × 79 μm, 3-μm particle size, Dikma, China) coupled to Easy nLC (Thermo Fisher Scientific, Waltham, MA). Peptides were eluted from 7% to 80% solvent B (0.1% formic acid in 90% acetonitrile and 10% H_2_O) in solvent A (0.1% formic acid in 2% acetonitrile and 98%H_2_O) with a 1 h gradient at a flow rate of 300 nl/min. The fractions were analyzed by using a Q Exactive mass spectrometer in a top16 data-dependent mode. For full MS spectra, the scan range was 350 to 1300 with a resolution of 70, 000. For MS/MS scan, the 16 most intense ions with charge state 2 and 3 in each full MS spectrum were sequentially fragmentated by higher energy collisional dissociation (HCD) with normalized collision energy of 28%. The resolution was 17, 500. The dynamic exclusion duration was set to be 60 s, and the isolation window was 1.5m/z. All MS raw files were analyzed by MaxQuant software (version 1.0.13.13) and Mascot software (version 2.1) against the database uniprot_Human to identify proteins.

### Real-time PCR analysis

Total RNA from tissues and cells was extracted using the RNA Isolation Kit (Takara, Dalian, China) according to the manufacturer's instructions. Quantitative real-time PCR was performed by using an Applied Biosystems 7300 Real-time PCR System and a TaqMan Universal PCR Master Mix. Expression of the KLF4, p21 and Cyclin B1 was normalized to that of the β-actin.

### Western blot

Cells were harvested and lysed with ice-cold lysis buffer (50 mM Tris-HCl, pH 7.4, 100 mM 2-Mercaptoethanol, 2% w/v SDS, 10% glycerol). After centrifugation at 10000 × g for 10 min at 4°C, proteins in the supernatants were quantified and separated by 10% SDS PAGE. Western blot assay was performed using anti-FBXO22, KLF4, p21, Cyclin B1, GFP and GAPDH antibodies (Abcam, USA). GAPDH was determined as a loading control

### BrdU and cell invasion assays

A cell proliferation enzyme-linked immunosorbent assay (BrdU kit; Beyotime) was used to analyze the incorporation of BrdU during DNA synthesis following the manufacturer's protocols. Absorbance was measured at 450 nm in the Spectra Max 190 ELISA reader (Molecular Devices, Sunnyvale, CA). For cell invasion assays, cells were analyzed using extracellular matrix-coated invasion chambers (Millipore, CA, USA), and quantitated with a colorimetric microplate reader at 570 nm, according to the manufacturer's instructions.

### Colony formation assay

Cells were seeded in a 6-well plate 48 hours posttransduction and cultured for 8 to 10 days at 37°C in 5% CO_2_. Cells were fixed with 4% paraformaldehyde in phosphate-buffered saline (PBS), washed twice with PBS, and stained with a crystal violet solution (1% crystal violet, 10% ethanol in water). Stained cells were washed thrice with water and counted by under an optical microscope.

### Statistical analysis

The data shown represent the mean ± standard error (SE) values of three independent experiments. Significance was analyzed using Student's *t*-test (*p < 0.05, **p < 0.01, ***p < 0.001).

## SUPPLEMENTARY FIGURES


