# Supplementary material for: F-box protein FBXO22 mediates polyubiquitination and degradation of KLF4 to promote hepatocellular carcinoma progression
Source: Oncotarget. 2015 May 28;6(26):22767–75. doi: 10.18632/oncotarget.4082 (PMC4673198; doi:10.18632/oncotarget.4082)
Supplement: Supplementary file 1 [file oncotarget-06-22767-s001.pdf]

SUPPLEMENTARY FIGURES

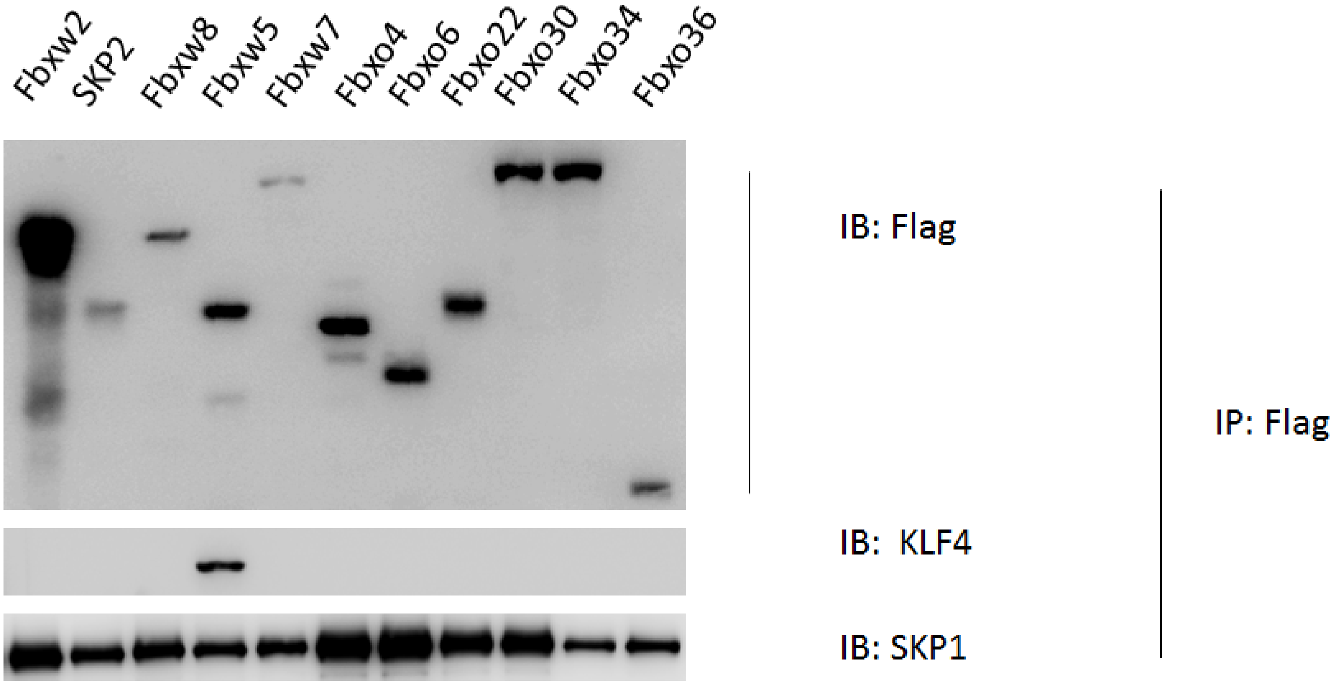

Supplementary Figure S1: The interaction of KLF4 and F-Box protein as indicated. HepG2 cells were transfected with adenovirus containing Flag-tagged F-Box proteins for 36 hr. Cells were harvested for immunoprecipitation with Flag-M2 beads and immunoblotted with antibodies against Flag, KLF4 and SKP1.

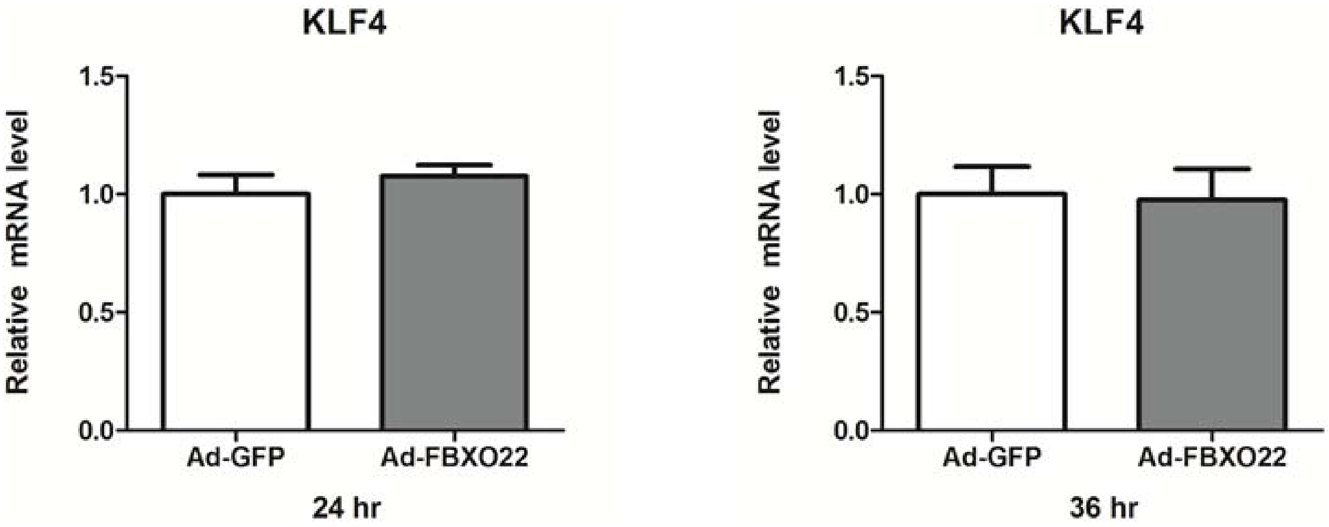

Supplementary Figure S2: Relative mRNA levels of KLF4 in HepG2 cells transfected with adenoviral shRNA targeting FBXO22 or a negative control (NC) for 24 or 36 hr.

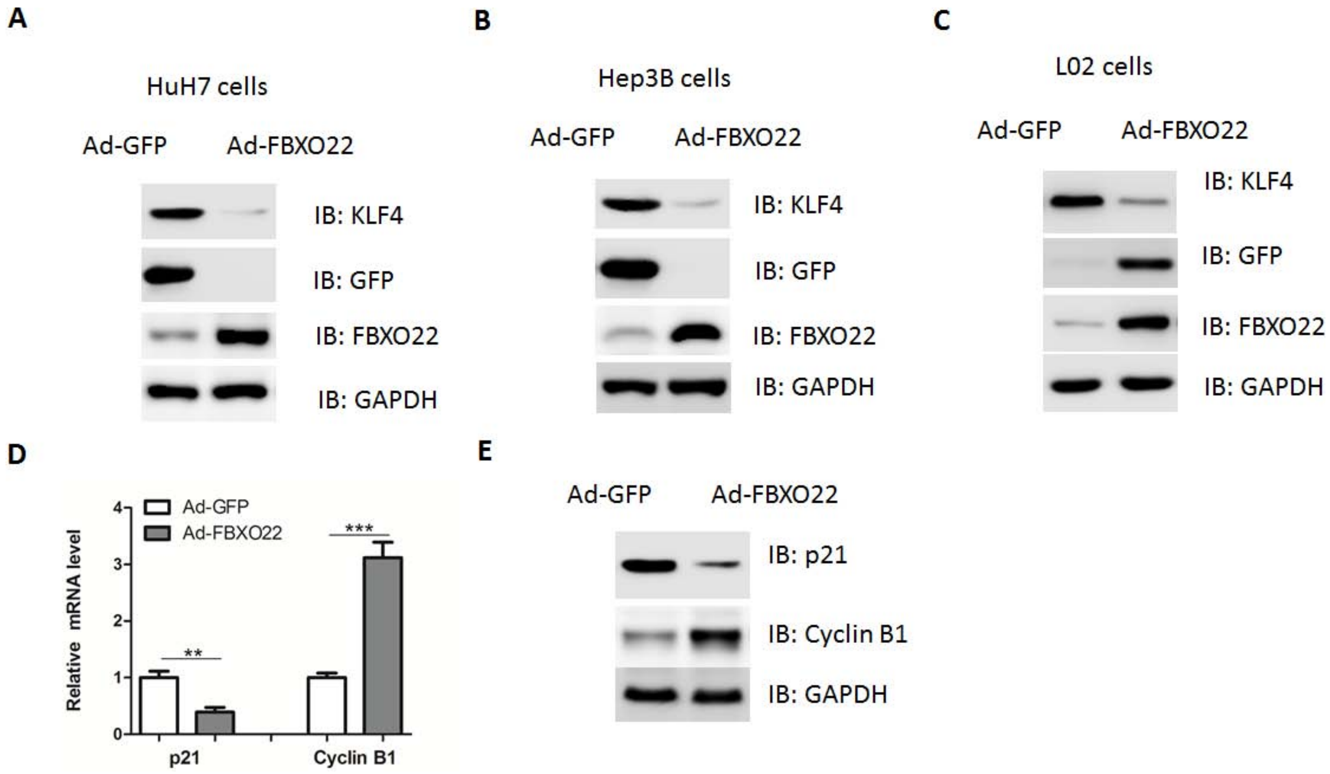

Supplementary Figure S3: A–C. The levels of KLF4 in cell lysates were determined by Western blot. HuH7 (A), Hep3B (B) or L02 (C) cells were transfected with adenovirus expressing GFP or FBXO22 for 36 hr. **D.** Relative mRNA levels of p21 and Cyclin B1 in HepG2 cells transfected with adenovirus expressing GFP or FBXO22 for 36 hr. **E.** Protein levels of p21 and Cyclin B1 in HepG2 cells were determined by Western blot.

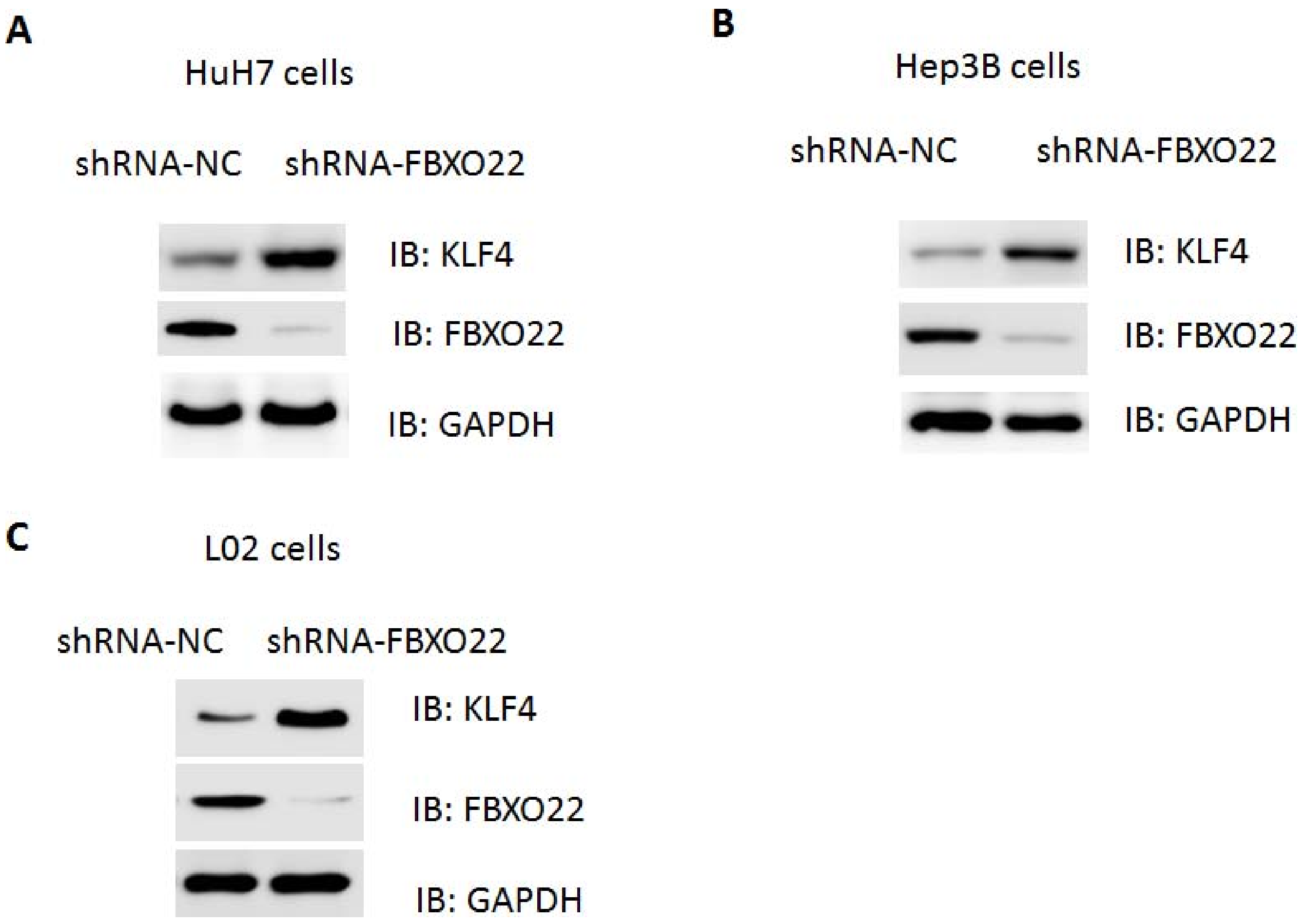

Supplementary Figure S4: A–C. The levels of KLF4 in cell lysates were determined by Western blot. HuH7 (A), Hep3B (B) or L02 (C) cells were transfected with adenoviral shRNA targeting FBXO22 or a negative control (NC) for 36 hr.

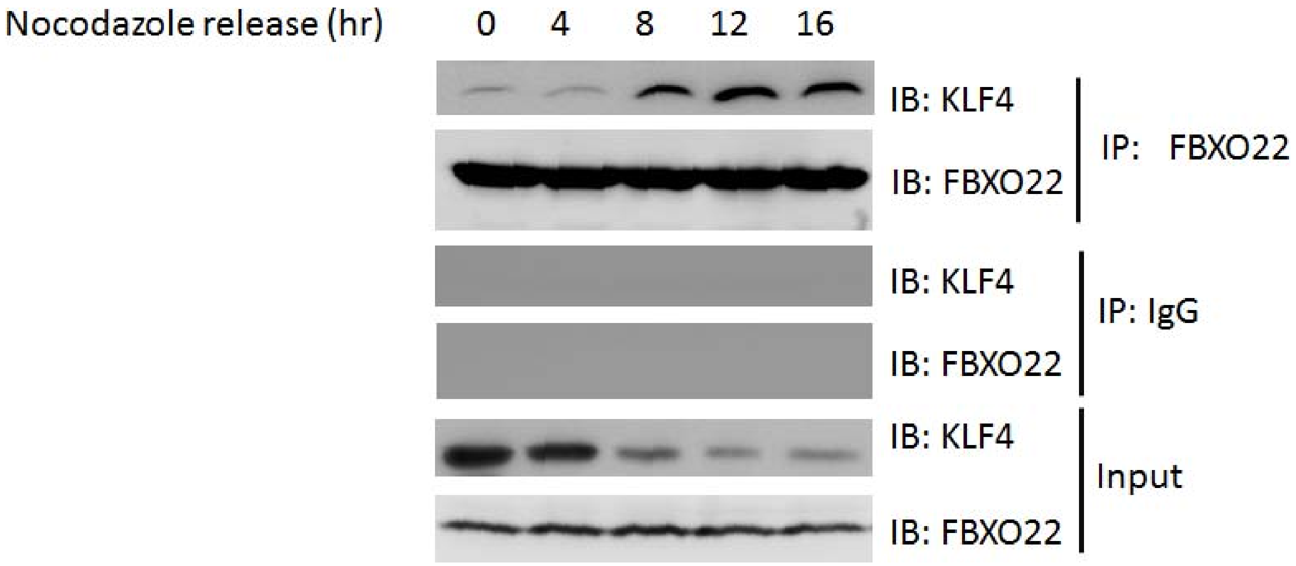

Supplementary Figure S5: Endogenous association between FBXO22 and KLF4 in HepG2 cells was performed by co-immunoprecipitation (CO-IP) experiments. Cells were synchronized by growth in nocodazole, and then released for the indicated periods.

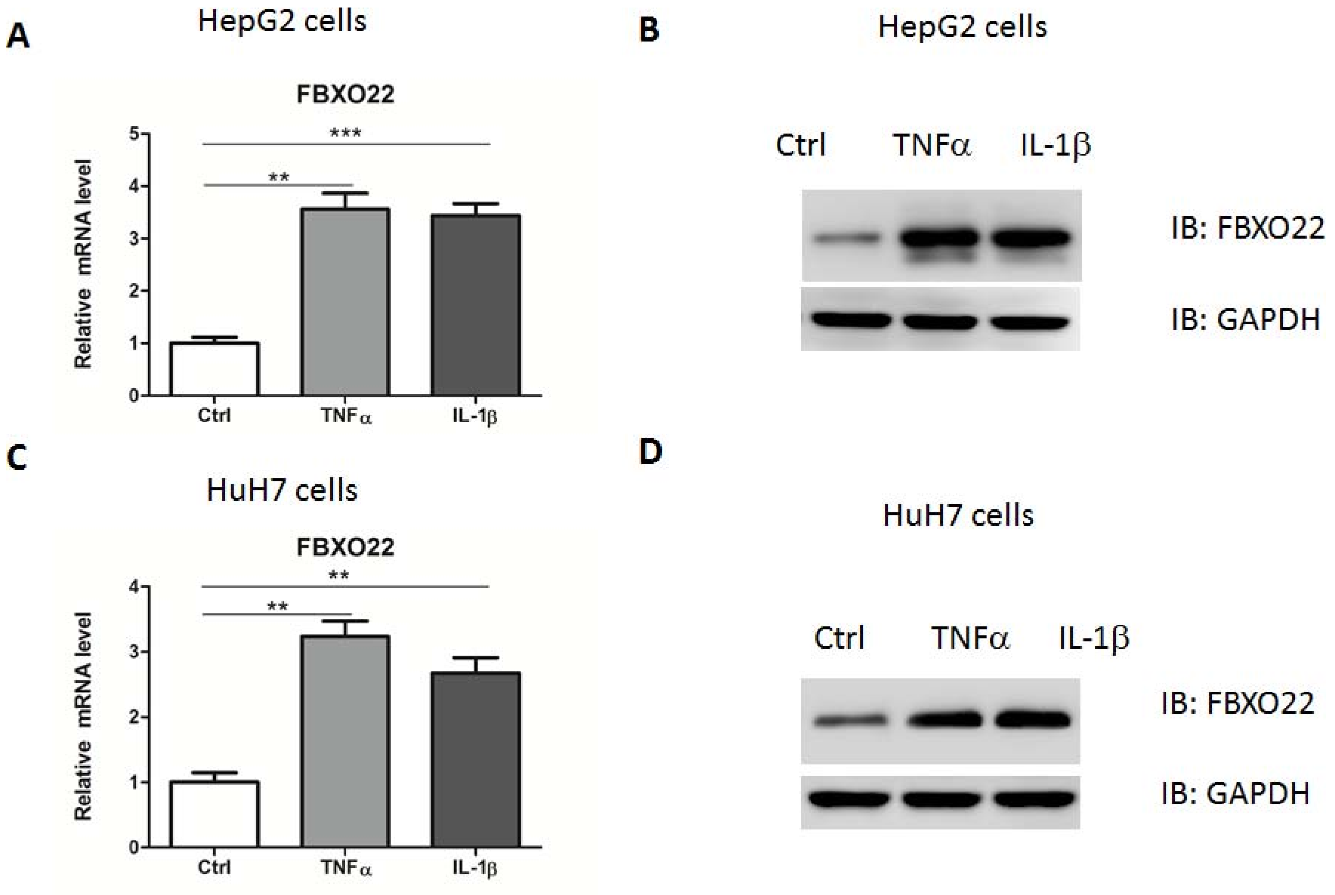

Supplementary Figure S6: A–B. mRNA (A) and protein (B) levels of FBXO22 in HepG2 cells treated with TNFα (10ng/ml), IL-1β (10ng/ml) or vehicle control for 24 and 36 hr, respectively. C–D. mRNA (C) and protein (D) levels of FBXO22 in HuH7 cells treated with TNFα, IL-1β or vehicle control for 24 and 36 hr, respectively.
